# Point mutation in D8C domain of Tamm-Horsfall protein/uromodulin in transgenic mice causes progressive renal damage and hyperuricemia

**DOI:** 10.1371/journal.pone.0186769

**Published:** 2017-11-16

**Authors:** Lijie Ma, Yan Liu, Nichole K. Landry, Tarek M. El-Achkar, John C. Lieske, Xue-Ru Wu

**Affiliations:** 1 Departments of Urology and Pathology, New York University School of Medicine, New York, New York, United States of America; 2 Division of Nephrology, Indiana University School of Medicine and Indianapolis VA, Indianapolis, Indiana, United States of America; 3 Division of Nephrology and Hypertension, Mayo Clinic, Rochester, Minnesota, United States of America; 4 Veterans Affairs New York Harbor Healthcare System, Manhattan Campus, New York, New York, United States of America; Hopital Tenon, FRANCE

## Abstract

Hereditary mutations in Tamm-Horsfall protein (THP/uromodulin) gene cause autosomal dominant kidney diseases characterized by juvenile-onset hyperuricemia, gout and progressive kidney failure, although the disease pathogenesis remains unclear. Here we show that targeted expression in transgenic mice of a mutation within the domain of 8 cysteines of THP in kidneys’ thick ascending limb (TAL) caused unfolded protein response in younger (1-month old) mice and apoptosis in older (12-month old) mice. While the young mice had urine concentration defects and polyuria, such defects progressively reversed in the older mice to marked oliguria, highly concentrated urine, fibrotic kidneys and reduced creatinine clearance. Both the young and the old transgenic mice had significantly higher serum uric acid and its catabolic product, allantoin, than age-matched wild-type mice. This THP mutation apparently caused primary defects in TAL by compromising the luminal translocation and reabsorptive functions of NKCC2 and ROMK and secondary responses in proximal tubules by upregulating NHE3 and URAT1. Our results strongly suggest that the progressive worsening of kidney functions reflects the accumulation of the deleterious effects of the misfolded mutant THP and the compensatory responses. Transgenic mice recapitulating human THP/uromodulin-associated kidney diseases could be used to elucidate their pathogenesis and test novel therapeutic strategies.

## Introduction

Of all the proteins present in the healthy human urine, Tamm-Horsfall protein (THP), also known as uromodulin, is by far the most abundant, with a daily urinary excretion rate of up to 100 milligrams [1–4]. The highly restricted expression of THP in the thick ascending limb (TAL) of the loop of Henle and the tendency of THP to self-aggregate and form a gel prompted earlier investigators to hypothesize that the protein contributes to the water impermeability of TAL [1,5]. Among other proposed functions for THP are modulation of urinary mineral crystallization, defense against bacterial infection, immune suppression, promotion of renal cast formation and stimulation of interstitial inflammation [1–3,6]. However, few of these proposed functions or disease roles were proven experimentally or clinically until relatively recently. Bleyer and colleagues were the first to demonstrate through genetic linkage studies that mutations in THP cause a group of hereditary hyperuricemic kidney diseases [7], a discovery later confirmed independently by other investigators [8–13]. These diseases that encompass several phenotypic variants such as familial juvenile hyperuricemic nephropathy, medullary cystic kidney disease type II and glomerular glomerulocystic kidney disease, share a common set of clinical manifestations including urine concentration defects, early-onset hyperuricemia, gout and end-stage renal disease (ESRD) around 50 years of age [14,15].

It has now been fairly well established that THP/uromodulin-associated diseases (UAKD) belong to endoplasmic reticulum (ER)-storage diseases. Like other proteins in this family, mutations of THP cause the protein to mis-fold and aggregate precociously, reducing its ability to exit the ER and translocate to the cell surface [11,15]. The mutated THP can also exert cytotoxic effects on the TAL cells and adversely affect the biosynthesis and functions of the host cell proteins. Preliminary evidence, based on clinical observations and expression of THP mutants in various cultured cells, suggests that the deleterious effects of THP mutations could be site-dependent, i.e., some mutations might cause more severe phenotypic alterations than others [16–19]. Of particular interest are the mutations within the so-called “domain of 8 cysteines” (D8C) [20]. D8C contains a stretch of 130 amino acid residues in THP and is highly homologous to the same domain present in liver-specific zona pellucida protein (LZP), pancreas-specific glycoprotein 2 (GP-2) and several uncharacterized proteins. The 8 highly conserved cysteines are predicted to form 4 pairs of disulfide bridges that are believed to be crucial for maintaining the correct conformational structures of the proteins [20]. Experimental verification to this prediction remains scare. When we previously compared a cysteine-altering mutation outside D8C (C126R) of THP with another inside it (C217G) in MDCK cells, we observed significantly greater deleterious effects of the latter in terms of ER-retention, reduced apical surface translocation, reduced release into culture media, apoptosis induction and entrapment of co-expressed wild-type THP [21]. Nonetheless, one could still argue that the phenotype could vary in intact animals as compared to cell-culture systems.

While two THP-mutant-expressing transgenic mouse models have already been developed, both involved mutations outside the D8C [22,23]. Given the increasing clinical evidence suggesting different severity of disease manifestations dependent on the mutation sites [16–19,21], it would be highly worthwhile to examine the in vivo effects of THP mutations involving the D8C domain. Additionally, analyses of the existing models have primarily focused on relatively young animals (up to 24 weeks or 6 months of age) [22,23] and, as a result, the progressive nature, one of the hallmarks of UAKD, has not been adequately recapitulated in mice. Finally, whether and precisely how THP mutations lead to hyperuricemia in transgenic models have not been assessed, probably due to the assumption that mice naturally make uricase, a uric acid degrading enzyme [24], and therefore that it would not be meaningful to measure uric acid in rodents. In the absence of direct experimental evidence, the tubular feedback between THP-caused TAL disturbance and the proximal tubule responses [13,25–27] remains somewhat speculative. The present study was therefore carried out to address some of these major issues and to gain a deeper understanding of the pathophysiology underlying UAKD.

## Materials and methods

### Generation and basic characterization of transgenic mice

A full-length mouse THP cDNA underwent a two-step site-directed mutagenesis to incorporate a hemagglutinin (HA) tag between amino acid residues 59 and 60 and to mutate codon 217 from cysteine to glycine (C217G) [21]. The cDNA was fully sequenced and tested extensively by transient and stable transfection in cultured cells for a wide range of effects as we published earlier [21]. For transgenic mouse work, the cDNA was fused to the 3’-end of a 3.0-kB mouse THP promoter that we isolated previously that was kidney- and TAL-specific [28,29]. The chimeric construct was again sequenced to ensure authenticity and the transgene was extracted from the cloning vector en bloc by restriction digestion. The restriction fragment was then purified and microinjected into one-cell embryos derived from an FVB/N inbred mouse strain for transgenic mouse production. Founder mice were identified by genomic PCR and confirmed by Southern blotting of restriction-digested genomic DNA from tail clipping. After germline transmission test, the founder bearing the highest number of the transgene was chosen to generate F1 progenies. Expression of the THP mutant in a kidney- and TAL-specific manner was established by a combination of RT-PCR, Western blotting, immunohistochemistry and immunofluorescent staining (see Methods below). Young and old mice were operationally defined as 1-month and 12-month old mice, respectively. Both male and female mice were used for analyses. All experiments involving the animals were conducted according to official policies and rules and after the approval of a protocol approved by the Institutional Animal Care and Use Committee of New York University School of Medicine.

### Measurement of 24-hour urine volume, urine chemistry and creatinine clearance

Randomly selected mice from both experimental and control groups were placed in single-mouse metabolic cages (Nalgene) supplemented with food and water ad libitum. Collection tubes contained 20 μl of 10% thymol and 10 μl of a Halt^™^ protease inhibitor cocktail (100mM AEBSF, 80 μM aprotinin, 5 mM bestatin, 1.5 mM E64, 2 mM leupeptin, 1 mM pepstatin A; ThermoScientific) and 10 μl of 0.5 M EDTA. At the conclusion of the 24-hour period, urine volumes were measured by pipetting and the samples were centrifuged at 500 x g for 5 min, and the supernatants were stored in a -80°C freezer until analysis. Urine chemistries were carried out at the Mayo Clinic O’Brien Urology Research Center Urinary Core using a Roche Cobas C311 autoanalyzer.

The concentrations of serum and urine creatinine from young and old WT and THP mutant mice were measured using a commercial kit (Biovision, Mountain View, CA). Mouse whole blood was obtained from the retro-orbital route with anesthesia (ketamine and xylazine), and the sera were obtained by centrifuging clotted blood at 2,000 x g for 10 min at 4°C. For creatinine assay, the serum and urine samples were diluted in an assay buffer and incubated in a solution containing creatinase, creatininase, enzyme mix and probe at 37°C for 1 h in 96-well microplates. The spectrophotometric readings at 570 nm were then plotted against a standard curve to derive the creatinine concentrations. The fractional creatinine clearance rate was calculated based on the following formula: urinary creatinine concentration × 24-h urine volume/serum creatinine concentration.

### Quantification of urinary THP

Urinary THP concentrations were measured from 24-hour urine samples using ELISA. Briefly, diluted urine samples were used to coat 96 well microtiter plates whose non-specific binding sites were then pre-blocked with 5% nonfat milk. Rabbit anti-THP (Courtesy of Dr. Franca Serafini-Cessi of University of Bologna, Italy) was used as primary antibody and peroxidase-conjugated goat anti-rabbit IgG was used as secondary antibody. TMB substrate and stop solution (0.16 M sulfuric acid; Thermo Scientific) were added consecutively and absorbance at 450 nm was read and plotted against a standard curve prepared using commercial THP standards (Accurate Chemical).

### Floatation assay of lipid raft

Total proteins were extracted from freshly dissected kidneys of WT and THP mutant mice (both 1-month of age) by homogenization and sonication in a neutral pH buffer containing 1% Triton X-100, 250 mM sucrose, 10 mM triethanolamine, plus a protease inhibitor cocktail detailed above. After centrifugation at 300 x g for 10 min at 4°C, the supernatants (1 mg per kidney) were transferred to the top of a stepwise sucrose gradient of 5–40% sucrose (in 5% increments) in 1% Triton X-100, 10 mM NaCl and 50 mM HEPES (pH 7.4). The entire assembly was centrifuged at 200,000 x g for 24 h at 4°C and 1 ml fractions were taken from every gradient interphase and the proteins (20 μl/fraction) were resolved by SDS-PAGE. Western blotting was then carried out using anti-NKCC2 antibody (Developmental Studies Hybridoma Bank; 1:5,000 dilution). The membrane was stripped and blotted again with an anti-flotillin antibody (Santa Cruz Biotechnology; 1:500 dilution).

### Confocal immunofluorescence microscopy, immunohistochemistry, immunoblotting and TUNEL assay

Formalin-fixed and paraffin-embedded kidneys were cut into 5-μm thick sections. Before staining, antigen retrieval was performed by microwaving deparaffinized sections in citrate buffer (pH 6.0). After pre-incubation of the sections in 5% BSA in PBS to block non-specific binding sites, the sections were subjected to single, double or triple staining with primary antibodies including sheep anti-THP (Novus; 1:200 dilution), rabbit anti-phospho-IRE1 (S-724) (Novus; 1:200 dilution), rabbit anti-BiP/GRP78 (Abcam; 1:500 dilution), rabbit anti-cleaved caspase 3 (Cell Signaling; 1:200 dilution), rabbit anti-Bax (Cell Signaling, 1:200 dilution), mouse anti-NKCC2 (Developmental Studies Hybridoma Bank, dilution 1:1,000), rabbit anti-ROMK (Novus; 1:50 dilution), rabbit anti-NHE3 (Novus; 1:100 dilution) or rabbit anti-URAT1 (Abbiotec; dilution 1:200). Corresponding species-specific secondary antibodies conjugated to either fluorescein or peroxidase were then used for immunofluorescent microscopy and immunohistochemistry, respectively.

For Western blotting, total kidney protein extracts from WT and THP mutant mice were resolved on SDS-PAGE and electrophoretically transferred to an Immobilon-PVDF membrane. After blocking of non-specific sites with 3% BSA in PBS, the membrane was incubated first with primary antibodies including anti-THP, anti-HA, anti-NHE3, anti-URAT1 and anti-β-actin and then with peroxidase-conjugated secondary antibodies. The membrane was developed in a chemiluminescent solution and exposed to X-ray film. For semi-quantification of URAT1 and NHE3, the specific, reactive bands were scanned using Image J software and the densities were normalized and expressed as ratios to β-actin. The average density from the WT mice was set at 1.

TUNEL assay was performed using an APO-BrdU^™^ Kit (Invitrogen) following the manufacturer’s instructions. Briefly, after paraffin sections were deparaffinized, they were incubated in a 20 μg/ml proteinase K solution for 15 min at room temperature and then in a TdT and BrdUTP solution for 60 min. After washing, the sections were incubated with anti-BrdU antibody conjugated with AF488 for 60 min at room temperature and counterstained with propidium iodide.

### Histopathology and histochemistry

Kidneys from WT and THP mutant mice were fixed in PBS-buffered 10% formaldehyde and embedded in paraffin. Four micron thick sections were cut, deparaffinized and stained with hematoxylin and eosin and examined with light microscope.

For detection of renal interstitial fibrosis, Mallory’s trichrome staining was employed on deparaffinized sections using a Trichrome Stain Kit (Sigma-Aldrich, St. Louis, MO) according to the manufacturer’s instructions. Areas containing the collagens that were stained blue and those without staining were imaged and calculated using an automated algorithm of color thresholding in NIH Image J software. Five fields per section at 200 x magnification were used for analysis and the data were expressed as the percentage of collagen deposition per 200 x field ± standard error.

### Determination of serum uric acid and allantoin

Whole blood was obtained from randomly selected WT and THP mutant mice via the retro-orbital route under anesthesia with ketamine and xylazine. Serum uric acid was determined using a commercial assay kit (BioVision, Milpitas, CA) following the manufacturer’s instructions. Serum allantoin was determined using a previously published method [30]. Briefly, 100 μl of serum per mouse was incubated with the allantoinase solution for 15 min at 37°C and the allantoate amidohydrolase solution for 10 min at 37°C. The readings of the reactions at 340 nm were plotted against a standard curve made with allantoin standards.

### Statistical analysis

Two-tailed student's *t*-tests were performed to evaluate the statistical significances between THP mutant mice and WT controls using Internet-based SPSS software. *P* values of less than 0.05 were considered statistically significant.

## Results

### Targeted expression of a THP mutation in D8C domain in the TAL of transgenic mice

**Fig 1:**
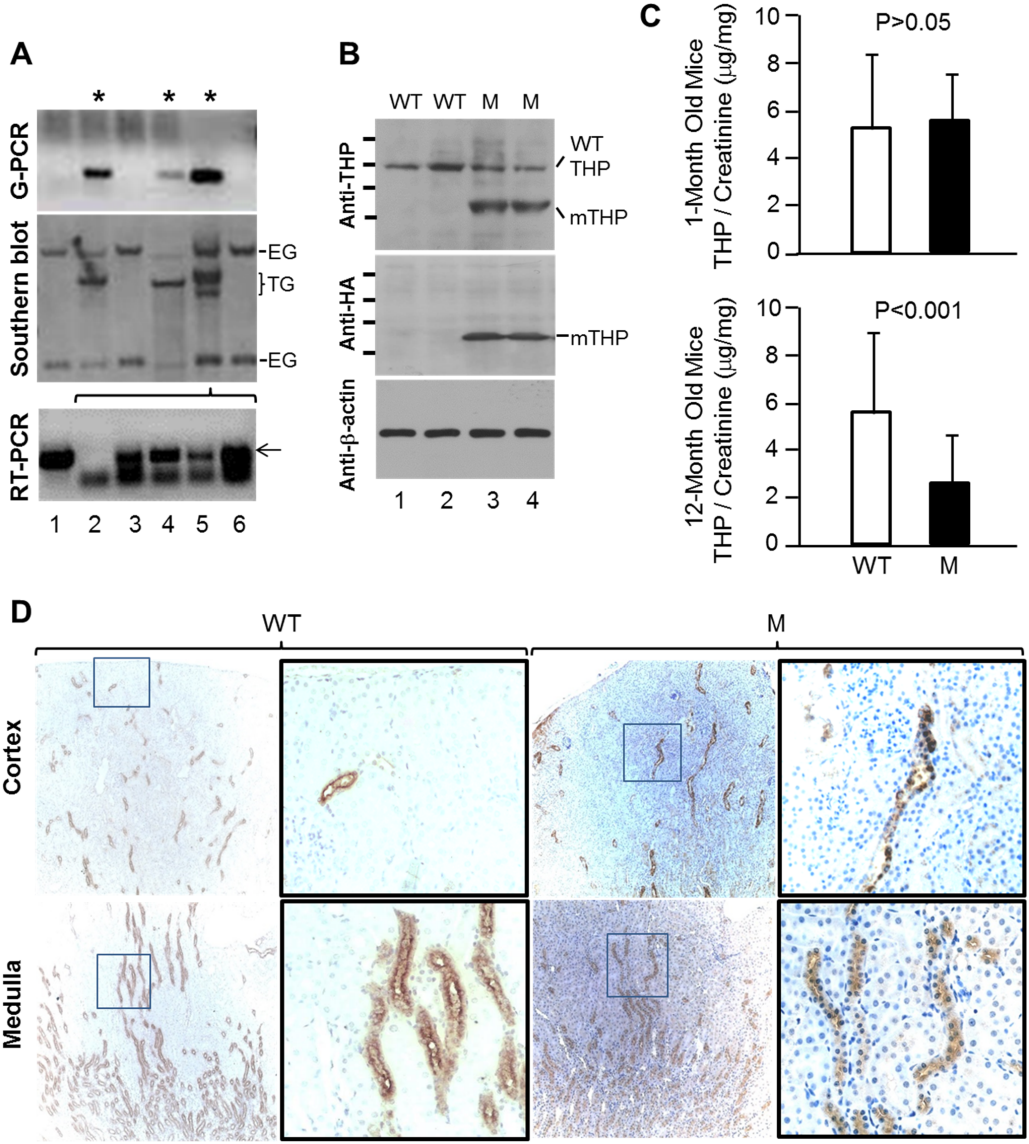
Generation and basic characterization of THP-C217G mutant mice. (A) Genomic PCR (G-PCR) and Southern blot analyses showing the generation of three founder mice (asterisks) that harbored a specific transgene (TG) fragment in addition to the two endogenous gene (EG) fragments, the latter of which were present in both non-founder and founder mice. One of these founder mice (lane 5) was chosen to generate F1 progenies whose kidneys upon RT-PCR analysis expressed a specific THP-C217G product (lanes 3–6, arrow) that matched in size to the product amplified from the THP-C217G-bearing plasmid (lane 1). Lane 2 was a negative PCR control. (B) Western blotting using anti-THP antibody of genotype-verified wild-type (WT) and THP-C217G mutant (M) mice showing the detection of the wild-type THP in all the mice and the smaller molecular-weight mutant THP-C217G (mTHP) only in the mutant mice. Mutant-specific anti-hemagglutinin (HA) antibody detected the smaller molecular-weight THP-C217G mutant species only in the mutant mice. Short bars on the left represented the molecular standards (from top to bottom: 135-, 100-, 72- and 55-kDa). Anti-β-actin served as a loading control. Gel and blot images in (A and B) were partially cropped to save space and their full-length versions are available upon request. (C) Detection of urinary THP by ELISA showing comparable levels between WT and THP-C217 mutant mice at young age (1-month) and marked reduction in old THP-C217G mutant mice (12-month), compared to age-matched WT mice. (D) Immunohistochemistry showing strong luminal and weak cytoplasmic staining of THP in WT mice (WT) and primarily cytoplasmic staining of THP in THP-C217G mutant in mice (M). Magnifications in (D), left two panels in WT and mutant mice, 50 x; and right two panels in WT and mutant mice (enlarged from the boxed insets in the left panels), 400 x.

In order to further our understanding of the pathophysiological consequences of THP mutations, we engineered a point mutation converting the first cysteine within the D8C domain of THP to glycine (C217G), a mutational switch common in patients with uromodulin-associated kidney diseases (UAKD) [9,31]. We also inserted a hemagglutinin (HA) tag between amino acid residues 59 and 60 to facilitate specific detection of the mutant THP and distinguish it from the endogenous wild-type THP [21]. To target the expression of THP-C217G specifically to the TAL cells where THP is normally synthesized, we fused the cDNA encoding mouse THP-C217G with a 3.0-kB mouse THP promoter we isolated and characterized earlier [21,28,29]. Of the three transgenic founder mice generated, we chose one that harbored the highest number of transgene copies for further analyses (Fig 1A, lane 5). Western blotting of the kidneys from the young (1-month old), transgene-containing progenies showed reduced levels (about 50% less) of endogenous THP (MW, ~100-kDa) and a strong, lower MW (~65-kDa) species corresponding to the mutant THP-C217G (Fig 1B). The latter species was confirmed to be THP-C217G because it specifically reacted with anti-HA antibody and it was absent from the WT littermates (Fig 1B). The fact that the THP-C217G had a MW of only ~65-kDa representing the molecular weight predicted from primary sequence of THP [32] indicated that the mutant lacked any complex-type glycosylation and was largely trapped in the ER. In addition, the reduced amount of the endogenous ~100-kDa THP in THP-C217G mice suggested that the endogenous wild-type THP was also somewhat trapped, probably due to the interaction with the THP-C217G mutant protein [21]. Although the urinary excretion of THP did not significantly differ in young mice between WT and THP-C217G mutant groups, it was significantly lower in old THP-C217 mice than in age-matched WT mice (Fig 1C). Immunohistochemistry showed that, while THP staining was strongly luminal in the WT mice, it was primarily cytoplasmic in THP-C217G mice (Fig 1D). Our results suggested that the THP-C217G was highly misfolded in the ER and the bulk of it could not efficiently exit ER and translocate to the luminal surface, and that neither the endogenous nor the THP-C217G could be efficiently excreted into the urine in old THP-C217G mice.

### THP-C217G caused unfolded protein response in young mice and apoptosis in dld mice

**Fig 2:**
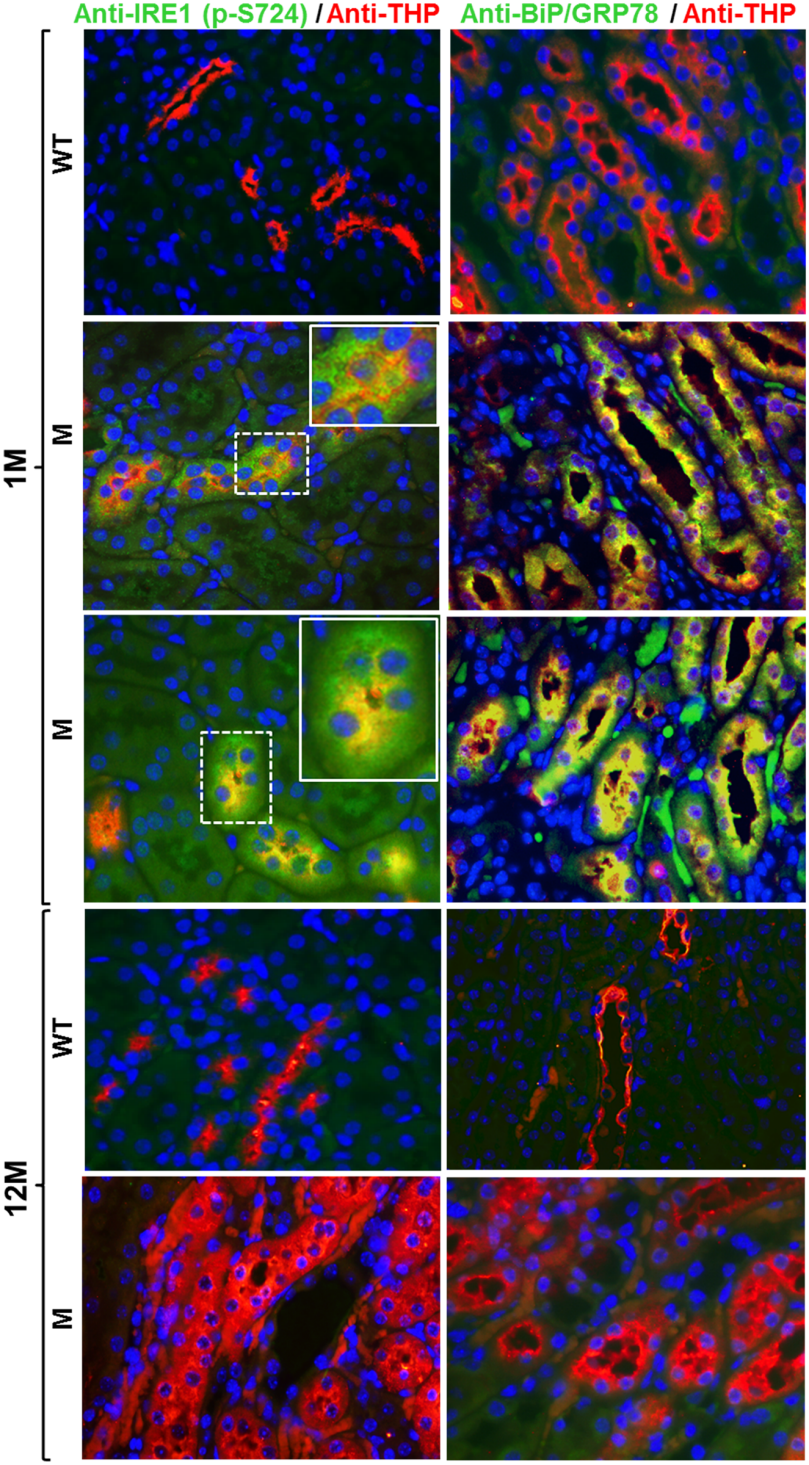
Assessment of unfolded protein response markers: Phosphorylated inositol-requiring kinase 1 (IRE1) and binding immunoglobulin protein/78-kDa glucose-regulated protein (BiP/GRP78). Double immunofluorescent staining of cross-sections of kidneys using anti-phospho-IRE1 (S724) and anti-THP (left column) or using anti-BiP/GRP78 (right column) and anti-THP-(merged double-staining images in both columns; yellow color denotes colocalization) showed that both phosphorylated IRE1and BiP/GRP78 were absent in tubular-cells (TAL cells) that were strongly labeled with anti-THP antibody in 1-month (1M) old WT mice (the first row). In contrast, phosphorylated IRE1 and BiP/GRP78 were highly expressed in the THP-positive TAL cells in age-matched THP-C217G mutant mice (the second and third rows). Insets (solid white line-boxes) showed higher magnifications of the areas in dash-lined boxes. However, phosphorylated IRE1 and BiP/GRP78 were undetectable in 12-month old (12M) WT mice (the fourth row) as well as in THP-C217G mutant mice (the fifth row). Nuclei were labeled blue by DAPI. Magnification for all panels, 400x and insets, 800x.

**Fig 3:**
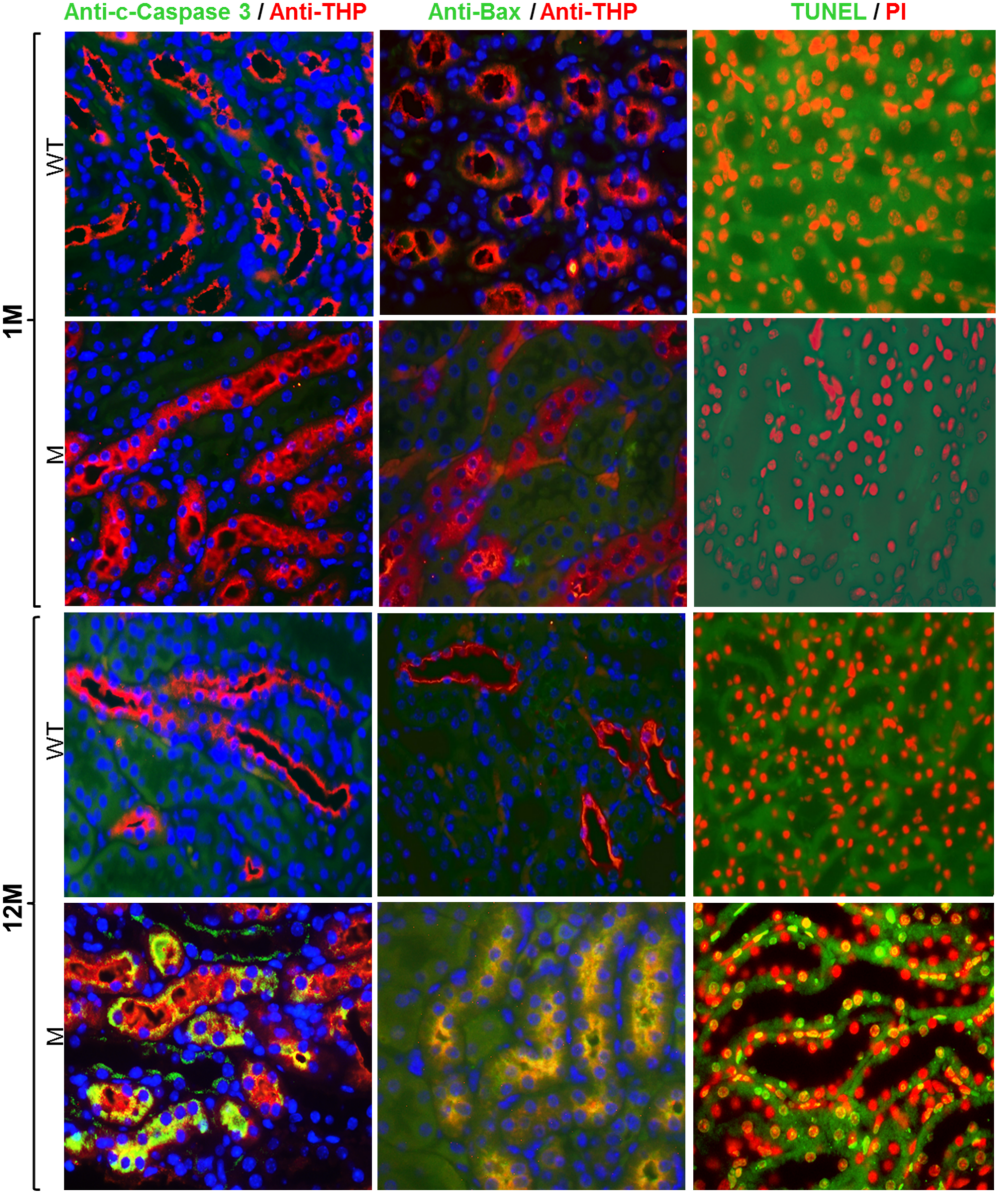
Assessment of apoptotic markers. Double immunofluorescent staining of cross-sections of kidneys (all merged images) showed that cleaved caspase 3 (c-caspase 3, Bax (middle column) and TUNEL labeling(right column) were all absent in 1-month old WT mice (1M) and THP-C217G mutant mice (the first and second row, respectively). While 12-month old WT mice (12M) also lacked all the three apoptotic markers (the third row), the age-matched THP-C217G mutant mice (M) had strong staining for these markers in THP-positive TAL cells (the fourth row). Nuclei were labeled blue by DAPI in the left and middle columns and they were labeled red by propidium iodide (PI) in the right column. Magnification for all the panels: 400x.

To determine the initial and latent responses of TAL cells to the highly misfolded THP-C217G, we stained the cross-sections of the kidneys from young and old THP-C217G mice with their age-matched WT mice as controls using double-immunofluorescent microscopy. We found phosphorylated inositol-requiring kinase 1 (IRE1) (Fig 2; left column) and binding immunoglobulin protein/78-kDa glucose-regulated protein (BiP/GRP78) (Fig 2; right column), highly conserved markers for unfolded protein response in mammals [5], were both strongly upregulated in THP-positive TAL cells of 1-month old THP-C217 mice, but not in WT controls (Fig 2). Interestingly, these two markers were no longer expressed in 12-month old THP-C217G mice (Fig 2), suggesting that TAL cells reacted to THP mutations via an unfolded protein response in a transient manner. By contrast, cleaved caspase 3, Bax and TUNEL assay, all indicative of apoptotic response [33], were completely absent in 1-month old THP-C217G mice, but were strongly upregulated in THP-positive TAL cells of 12-month old THP-C217G mice (Fig 3).

### Interstitial fibrosis in the kidneys of THP-C217G mice

**Fig 4:**
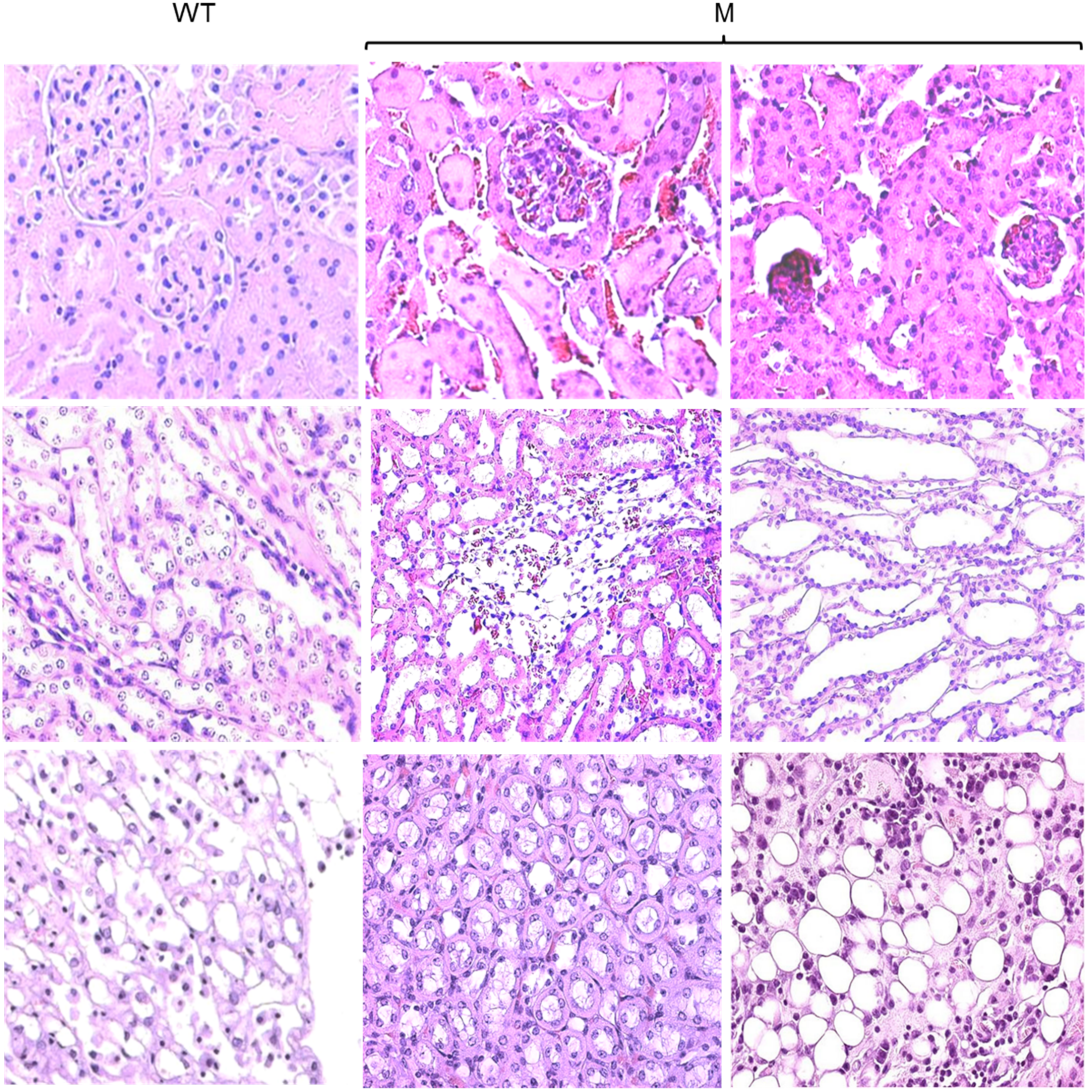
Histopathological features of 12-month old WT mice and age-matched THP-C217G mutant mice (M). Representative images of the cortex (the first row), medulla (the second row) and papillary (the third row) regions of the kidneys from 12-month old WT and age-matched THP-C217G mutant (M) mice after routine paraffin-sectioning and H&E staining. Note the normal renal morphology in the WT mice and a series of pathological changes in the THP-C218G mutant mice including sclerotic and shrunk glomeruli, tubular atrophy, enlarged tubular lumen, interstitial fibrosis and fatty degeneration. Magnification for all the panels: 400x.

**Fig 5:**
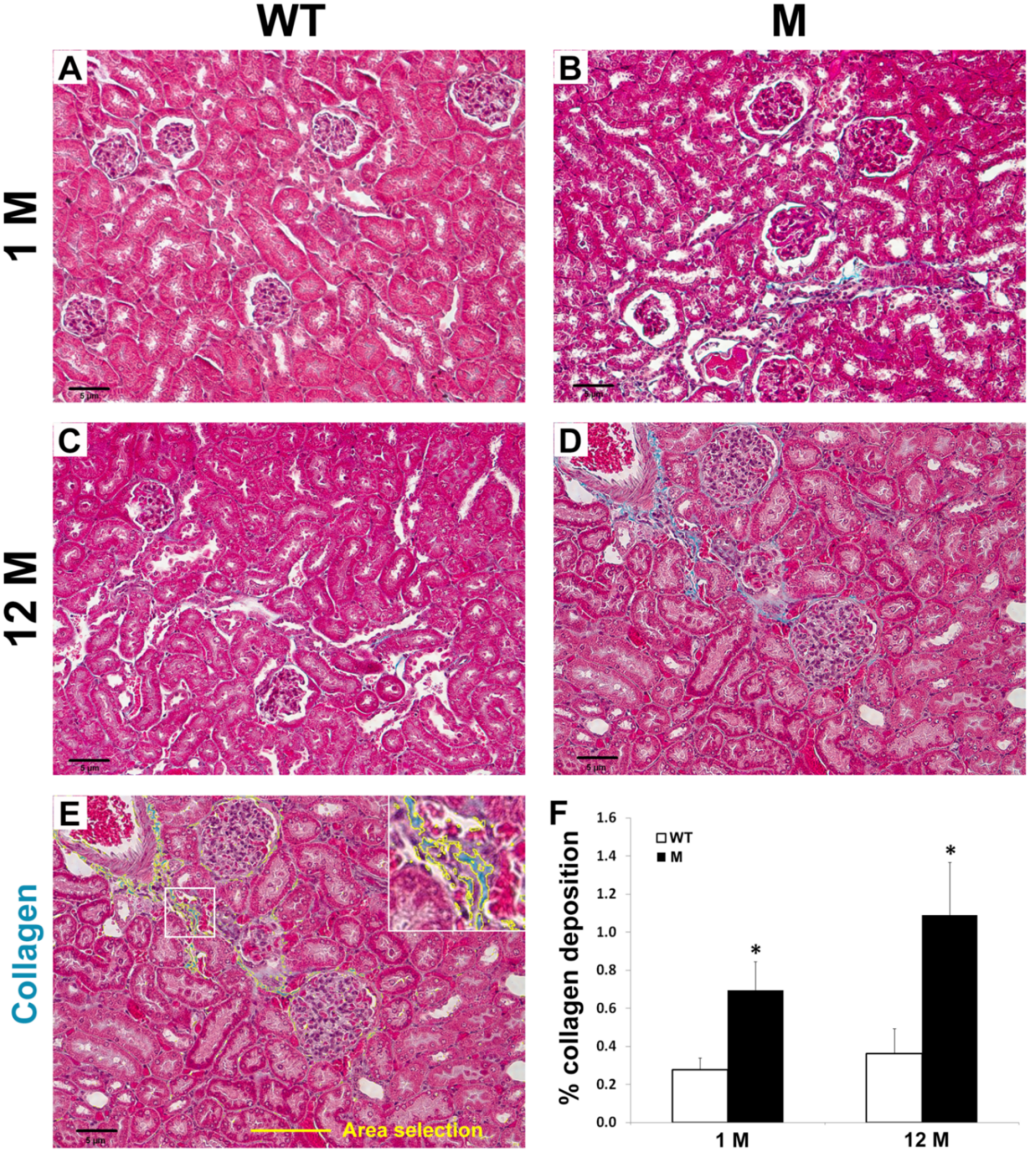
Histochemical staining and semi-quantitation of tubulo-interstitial fibrosis in young and old WT and THP-mutant mice. (A-D) Representative images of light microscopic fields (200 x) from cortical kidney sections stained with Mallory’s trichrome. The mice were grouped into wild-type (WT; A and C) vs. mutant (M; B and D)) at 1 month (1M) vs. 12 months (12M) of age (n = 7 per group). (E) Five fields per section were used for analysis and the area percentage of collagen deposition was calculated using an automated algorithm of color thresholding in Image J. The selection highlighted in yellow (see enlarged inset of the area outlined by the white box) represented the area that was calculated for analysis. (F) The bar graph represented means ± SE of the percentage of collagen deposition per 200 x field. Asterisks denote statistical significance (p<0.05) between WT and M.

To determine whether the apoptosis of the TAL cells expressing the THP-C217G would lead to pathological changes in the kidney of the THP-C217G mice, we first stained cross-sections of kidneys the old mice by H&E (Fig 4). While, as expected, the old WT mice were devoid of overt pathological changes, old THP-C217G mice exhibited extensive atrophic tubules and interstitial fibrosis in medullary and papillary regions. Sclerotic glomeruli were also present in the cortex (Fig 4). We next performed Malloy’s trichrome staining and semi-quantification to assess the extent of collagen deposition in both young and old mice (Fig 5). We found increased collagen deposition in the young THP-C217G mutant mice and more so in old THP-C217G mutant mice than their age-matched WT controls (Fig 5).

### Progressive urine volume and chemistry alterations in mice expressing THP-C217G

**Fig 6:**
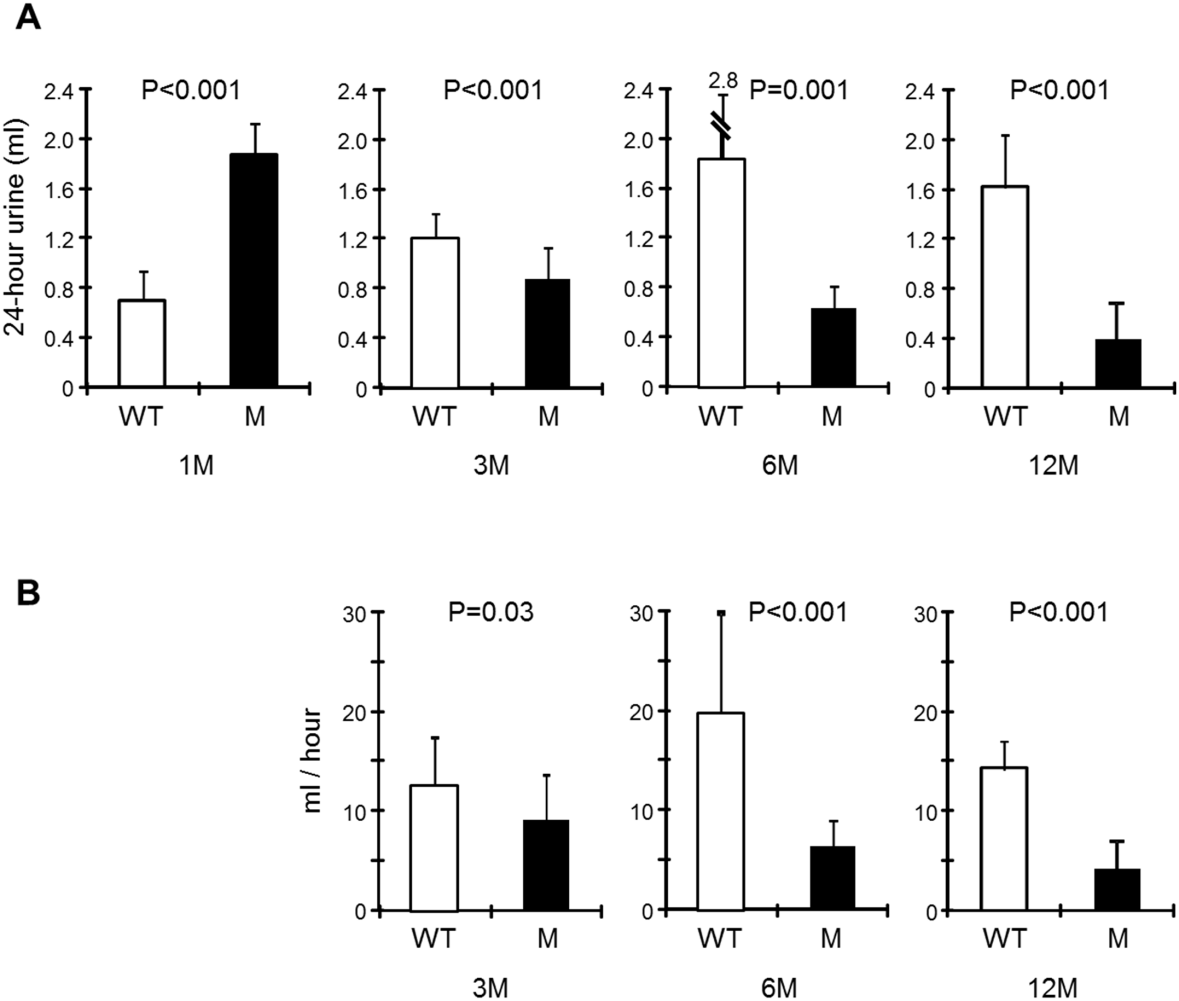
Progressive urine volume changes (A) and creatinine clearance (B) in THP-C217G mutant mice. (A) WT (n = 11/age group) and THP mutant mice (n = 9/age group) at 1, 3, 6 and 12 months of age (abbreviated as 1M, 3M, 6M and 12M) were subject to 24-hour urine volume measurement in single-mouse metabolic cages. Note the significantly higher urine volume in 1-month old THP-C217G mutant mice (compared to age-matched WT mice) and the progressive reduction of urine volume in THP-C217G mutant mice with the increasing age. (B) Creatinine clearance of WT and THP mutant mice at 3, 6 and 12 months of age, showing progressive reduction in aging THP mutant mice, as compared to age-matched WT mice.

Because TAL is a crucially important nephron segment in maintaining water and salt balance [34], we determined the 24-hour urine volume in THP-C217G at 1, 3, 6 and 12 months of age with their age-matched WT mice as controls. We found the urine volume of 1-month old THP-C217G mice to be more than two-fold higher than that of 1-month old WT mice (Fig 6A). This trend was, however, reversed as early as 3-months. By 6 months, THP-C217G mice had about 1/3 of the urine output of the WT controls. At 12-months, THP-C217G mice had only about 1/5 of the urine volume of the age-matched WT mice (Fig 6A). Urine chemistry analysis showed that, compared with the WT mice, the 1-month old THP-C217G mice had much more diluted urine with significantly lower osmolality and lower concentrations of all the electrolytes including Na, Cl and K (Table 1). In stark contrast, compared to the WT mice, 12-month old THP-C217G mice had much higher urine osmolality and lower 24-hour excretion of all the electrolytes (Table 1). The creatinine clearance rate was also impacted with the advancing age in THP-C217G mice, with 6-month and 12-month old mice exhibiting significantly lower values than their age-matched controls (Fig 6B). Measurement of creatinine clearance in 1-month old mice was not possible due to the extremely small blood volume.

**Table 1:** Urine chemistry of young (1-month old) and old (12-month old) WT and THP-C217G mutant mice.

	1-month old mice		12-month old mice	
	WT (n = 20)	THP-mutant (n = 20)	WT (n = 20)	THP-mutant (n = 20)
Osmo (mOsm/kg)	2909.85 (813.38)	1765.20 (403.12)*	1731.30 (606.21)	2551.60 (606.02)*
Sodium (meq)	0.13 (0.04)	0.23 (0.06)*	0.20 (0.09)	0.09 (0.03)*
Chloride (meq)	0.19 (0.07)	0.29 (0.08)*	0.22 (0.09)	0.09 (0.04)*
Potassium (meq)	0.27 (0.10)	0.45 (0.16)*	0.36 (0.19)	0.15 (0.05)*
Phosphorus (mg)	2.47 (1.05)	3.57 (1.07)*	3.46 (1.36)	2.02 (0.63)*
Citrate (mg)	3.23 (1.93)	4.83 (2.41)*	2.59 (1.84)	1.36 (0.68)*
Calcium (mg)	0.09 (0.06)	0.13 (0.06)*	0.13 (0.08)	0.06 (0.03)*
Magnesium (mg)	0.41 (0.18)	0.65 (0.22)*	0.72 (0.33)	0.39 (0.12)*
Uric acid (mg)	0.12 (0.06)	0.16 (0.09)	0.15 (0.10)	0.07 (0.03)*
Creatinine (mg)	0.25 (0.09)	0.43 (0.13)*	0.52 (0.26)	0.31 (0.09)*
pH	6.22 (0.24)	6.39 (0.12)*	6.36 (0.49)	6.17 (0.11)

Values shown were averages of the 24-hour total excretion and standard deviations (in parentheses).

* marked comparisons within an age group with statistical significance (p<0.05) between WT and THP-C217G mutant mice.

### THP-C217G adversely affected the lipid raft partitioning and luminal translocation of major ion transporters in the TAL

**Fig 7:**
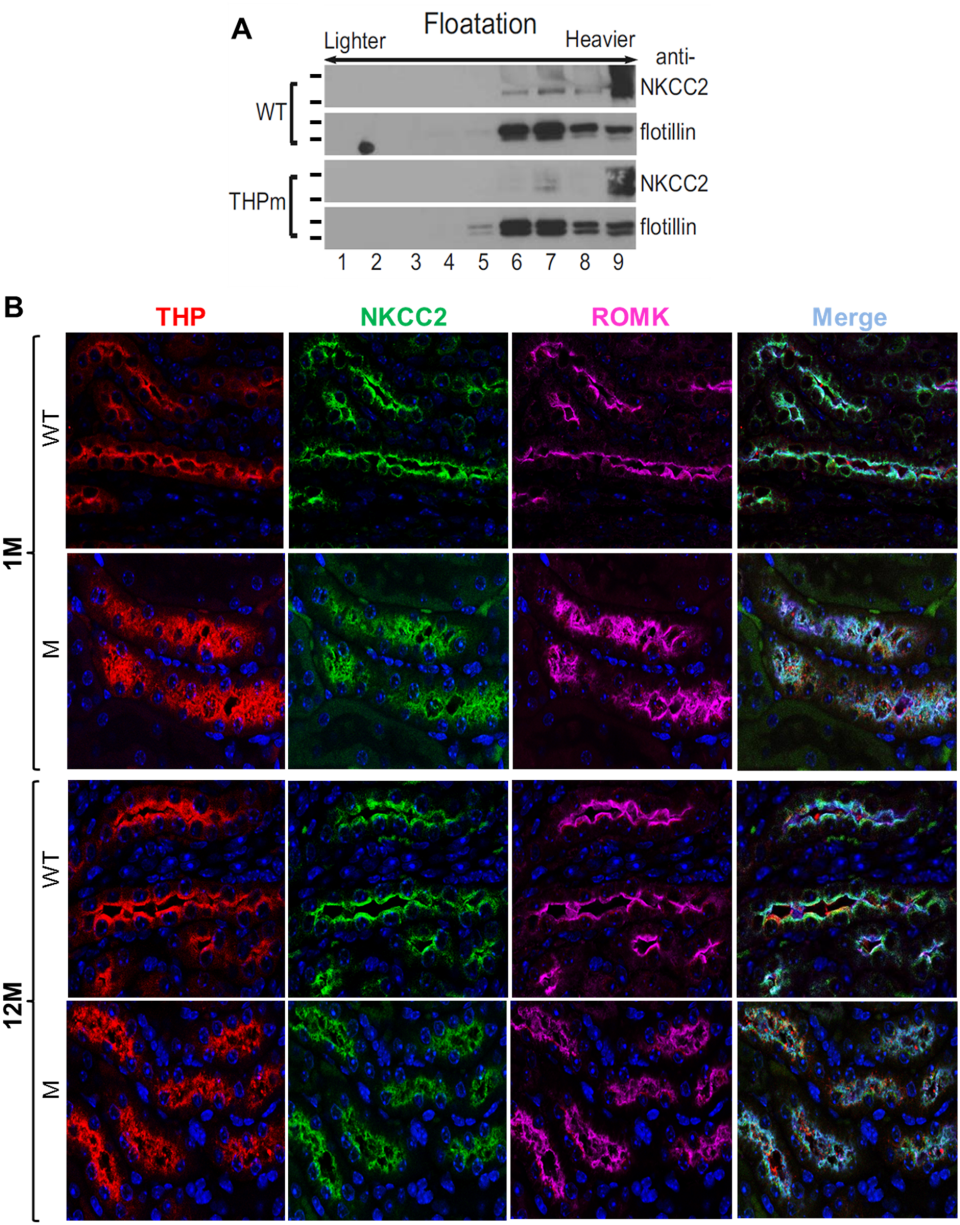
Partitioning in lipid raft and localization of key TAL transporters. (A) Partitioning of NKCC2 in the lipid rafts by flotation assay. Freshly dissected kidneys from 1-month old WT and THP-C217G mutant mice were homogenized in Triton X-100 and the-insoluble proteins were resuspended in the same solution and centrifuged on a sucrose gradient (5–40% with 5% increments), after which equal volumes from fractions 1 through 9 (lighter-density fractions on the left and heavier-density fractions on the right) were subject to SDS-PAGE and immunoblotting with anti-NKCC2 and anti-flotillin. Note that NKCC2 was present in the lighter fractions (fractions 6 and 7) in WT mice but was reduced or absent in the lighter fractions in THP-C217G mutant mice. The short bars left to the blots denote the molecular weight standards (for NKCC2 blots from top to bottom: 170- and 135-kDa; for flotillin blots (55- and 40-kDa). Western blot images were cropped to save space and their full-length versions are available upon request. (B) Triple immunofluorescent staining of THP, NKCC2 and ROMK in the TAL of WT and THP-C217G mutant mice. Note the almost exclusive luminal staining of THP, NKCC2 and ROMK in 1-month old WT mice (the first row), the partial luminal staining and increased cytoplasmic staining of all the three proteins in 1-month old THP-C217G mutant mice (the second row), the strong luminal staining of all the three proteins in 12-month old WT mice (the third row), and the marked reduction of luminal staining and marked increase of cytoplasmic of all the three proteins in 12-month old THP-C217G mutant mice (the fourth row). Magnification: 400 x.

Because ion transporters such as NKCC2 and ROMK are normally expressed in the TAL [35,36] and their expression might be adversely affected by the expression of a THP mutant, we assessed the status of NKCC2 in young THP-C217G mice by lipid raft floatation assay and the localization of NKCC2 and ROMK in conjunction with THP by triple immunofluorescent staining in both young and old THP-C217G mice (Fig 7). We found NKCC2 to be primarily partitioned in the heaviest fraction (fraction 9) with lighter fractions (fractions 6–8) largely devoid of NKCC2, as compared with the WT mice (Fig 7A). This result was confirmed independently by triple immunofluorescent staining. Specifically, in young WT mice, NKCC2 and ROMK exhibited an excellent luminal labeling along with that of THP (Fig 7B). A decreased luminal labeling and increased cytoplasmic labeling was evident in 1-month old THP-C217G mice. Old WT mice maintained the luminal polarity of NKCC2, ROMK and THP. However, all the three proteins were almost completely cytoplasmic in old THP-C217G mice (Fig 7B).

### Elevated serum uric acid and allantoin and increased expression of URAT1 and NHE3 in THP-C217G mice

**Fig 8:**
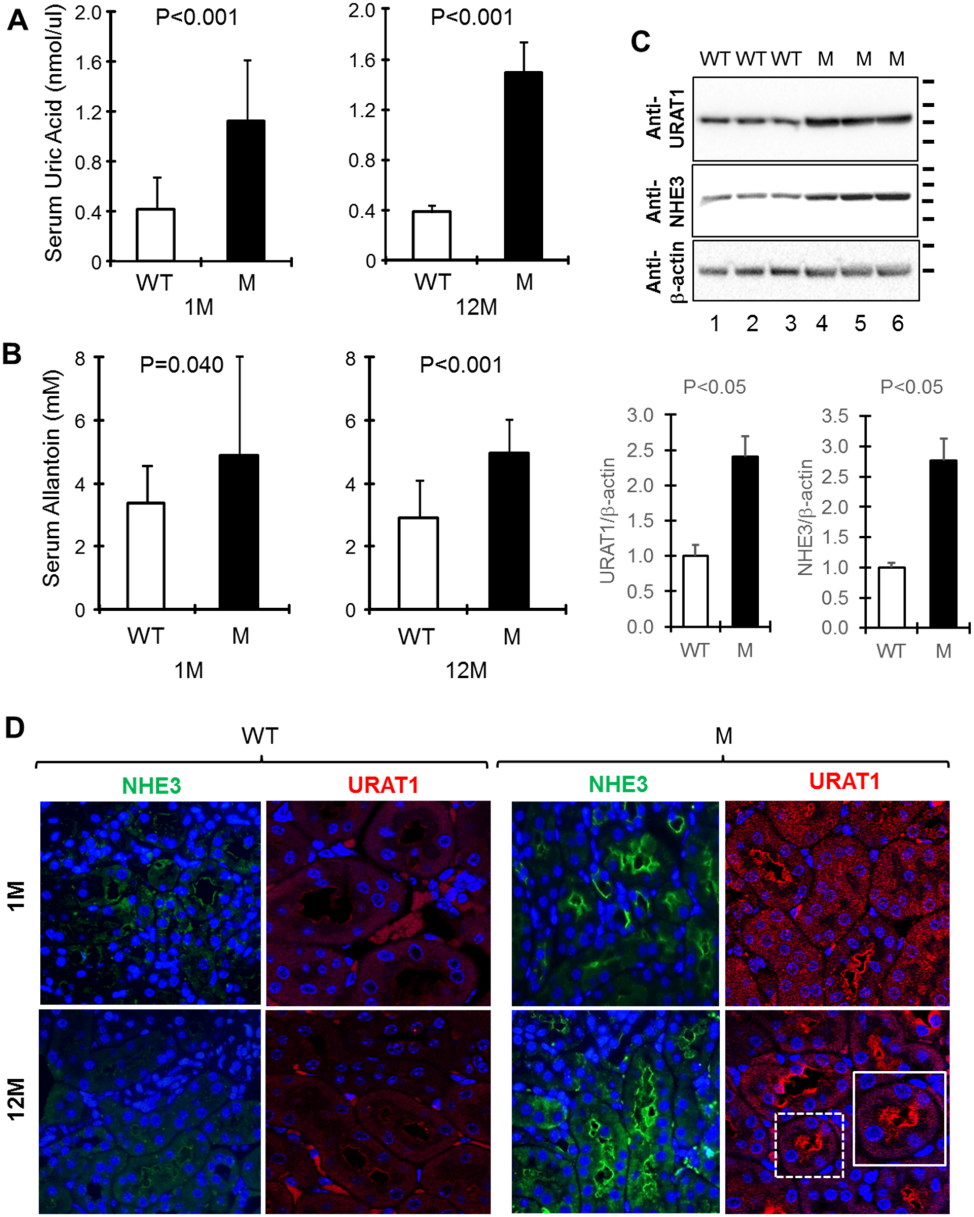
Serum levels of uric acid and allantoin and expression of sodium/hydrogen exchanger and urate transporter. (A and B) 1- and 12-month old WT and THP-C217G mutant mice were subject to measurement of serum uric acid and allantoin (n = 20/group). Note the significantly higher levels of serum uric acid and allantoin in both young and old THP-C217G mutant mice than in their age-matched WT mice. (C) Western blotting detection (upper panels) and semi-quantification (lower panels) of urate transporter 1 (URAT1) and sodium–hydrogen exchanger 3 (NHE3) in 12-month old WT and THP-C217G mutant mice (3 mice each) showing significantly increased levels of both proteins in the mutant mice. The short bars right to the blots denote the molecular weight standards (for URAT1 blot from top to bottom: 100-, 72-, 55- and 40-kDa; for NHE3 blot: 135-, 100-, 72- and 55-kDa; and for β–actin blot: 55- and 40-kDa). (D) Double immunofluorescent staining of NHE3 and URAT1. Note the increased levels of NHE3 in 1- and 12-month old THP-C217G mutant mice, compared with their age-matched WT counterparts. Also note the increased expression of URAT1 in both 1-month and 12-month old THP-C217G mutant mice, compared with their age-matched WT controls. Magnification for all panels in C, 400x and inset, 800x.

Although patients with THP mutations often experience hyperuricemia [8,9,15], this key clinical manifestation has not been reproduced in transgenic mouse models expressing human-relevant mutations [22,23]. This probably could be attributed to the assumption that mice synthesize a functional uricase [24] and that measuring their uric acids would yield false-negative results. When we nevertheless measured serum uric acid, we found that its levels were significantly higher in both young and old THP-C217G mice compared to their age-matched controls (Fig 8A). Consistent with this, the serum levels of allantoin, the catabolic product of uric acid, were also significantly elevated in young and old THP-C217G mice (Fig 8B).

Since the bulk of uric acid reabsorption takes place in the proximal tubules and is functionally coupled with Na and proton exchange [25–27], we assessed the levels of sodium–hydrogen antiporter 3 (NHE3) and urate transporter 1 (URAT1) [26,37,38,39] using Western blotting (Fig 8C) and immunofluorescent staining (Fig 8D). We found that both NHE3 and URAT1 were upregulated by about 2.5 fold in the mutant mice compared to the WT mice, as evidenced by Western blotting and semi-quantification (Fig 8C). This upregulation and apical localization of NHE3 and URAT1 in young and old THP mutant mice was also evident by immunofluorescent staining (Fig 8D). Together, these results raised the possibility that the TAL dysfunction due to THP mutation triggered compensatory responses in the proximal tubules, leading to hyperuricemia.

## Discussion

Genetically engineered mouse models are powerful tools for defining the molecular and cellular mechanisms of genetic diseases and for evaluating new diagnostic and therapeutic approaches. There are, however, only two models in existence that explored the pathogenesis of THP/uromodulin-associated diseases (UAKD) [22,23]. Both models targeted the expression of human-relevant C148W mutation of THP into the thick ascending limb (TAL) of loop of Henle of the kidney under the direction of a 3.0-kB THP gene promoter, with somewhat divergent results. The model developed by Takiue and coworkers expressed a human THP cDNA harboring C148W [22]. While there was an accumulation of THP in the TAL, the authors reported that the urinary THP was primarily of the mouse origin and its level was not lower than that of the non-transgenic controls [22,23]. This raised the possibility that the mutant THP did not have an obvious dominant-negative effect on the endogenous wild-type THP. Additionally, none of the clinical manifestations associated with human UAKD was observed in these transgenic mice up to 24 weeks of age [23]. Another model developed by Rampoldi’s group expressed an isogenic mouse THP cDNA harboring a C147W mutant, which is equivalent to human C148W [23]. As opposed to the lack of phenotype in the Takiue model [22], the Rampoldi model exhibited urinary concentrating defect, tubulointerstitial fibrosis and mild renal failure by 24 weeks of age [23]. These authors later showed that the C147W THP mutant could partially exit the ER and reach the cell surface to exert a dominant-negative effect on the endogenous, wild-type THP [40]. Since both models made use of the 3.0-kB THP promoter, it is unlikely that the divergent results of the same mutation could be attributed to the promoter activity per se. It cannot be completely ruled out, however, that due to sequence divergence, the C148W-bearing human THP in the Takiue model [22] had different functional attributes than the C147-bearing isogenic mouse THP in the Rampoldi model [23].

In our present study, we engineered a C217G mutation by site-mutagenesis in isogenic mouse THP cDNA and expressed it in transgenic mice under the control of the 3.0-kB mouse THP promoter we isolated earlier [28,29]. This mutation is human-relevant as it occurs in patients with UAKD [9,31]. It affects the first cysteine within the D8C domain and therefore differs from the previously engineered C148W which is located outside D8C [20]. Our previous study in cultured cells demonstrated that the D8C mutant C217G exerts significantly greater deleterious effects in ER-retention, reduced apical surface translocation, reduced release into culture media, apoptosis induction and entrapment of co-expressed wild-type THP than the non-D8C mutant C126R [21]. This prompted us to carry out the *in vivo* transgenic work. We found our model to share several features with the Rampoldi’s model [23] including the urine concentration defect (Table 1; Fig 6A), interstitial fibrosis (Figs 4 and 5) and renal insufficiency (Table 1; Fig 6). Beyond these, we observed additional phenotypic alterations that were not observed before, including (i) the progressive changes of TAL cells from unfolded protein response in young mice to marked apoptosis in old mice (Figs 2 and 3), (ii) the major defects in the apical translocation of key TAL transporters NKCC2 and ROMK (Fig 7), (iii) the severe oliguria resembling ESRD in old mice (Table 1; Fig 6), and (iv) the hyperuricemia in both young and old mice and the overexpression of both NHE3 and URAT1 in the proximal tubules (Fig 8).

The phenotypic spectrum of our model expressing the THP-C217G is therefore more broad and severe than those of the previous two models and, our model in several respects, more closely resemble the manifestations of the UAKD patients. The higher degree of phenotypic severity observed in our mice may have to do with the longer follow-up (48 weeks in our cohorts which is twice as long as that in the previous cohorts; [22,23]). It could also be due to the fact that C217G resides in D8C [20] and causes more profound conformational abnormalities of THP. Data from our cell culture studies and the present transgenic mice showing that the C217G THP mutant is entirely trapped in the ER support this possibility [21]. While ideally one should include a non-D8C mutant in the transgenic work for comparative purposes, we had to forego such a design because of high cost concerns. Age difference might not be a distinguishing factor since hyperuricemia was already prominent in our 1-month old transgenics before any apparent structural abnormality (Fig 8). Functional, rather than structural, causes linked to the reduced absorptive functions of TAL seem implicated. As evidenced by lipid raft flotation assay and triple immunofluorescent confocal microscopy (Fig 7), C217G apparently caused a marked reduction of partitioning in lipid raft and luminal translocation of NKCC2 and ROMK, two key transporters of Na, Cl and K in the TAL. This explains the salt wasting and urine concentration defects in our young transgenic mice (Fig 6) and is consistent with the increased excretion of urinary Na, Cl, Ca, Mg, increased urine pH and decreased urinary osmolality. Because the weights of young THP mutant mice do not differ significantly from those of the young wild-type controls (data not shown), the increased urine creatinine may have to do with an increased food intake as a compensatory mechanism to increased urinary loss. It should be noted that mice lacking THP in the KO mice leads to an almost identical outcome to NKCC2 [41], suggesting a strong parallel between THP mutant mice and THP KO mice.

What then underlies the hyperuricemia in our C217G THP mutant mice? The answer may lie in the compensatory responses of the proximal tubules as recently envisioned by other investigators in the field [13,25–27]. Urine concentration defects and salt loss due to NKCC2 and ROMK mislocation in the TAL may trigger a compensatory increase of Na and Cl uptake from the proximal tubules. This could take place in two functionally coupled steps: (i) upregulation of Na-proton exchanger and (ii) increased expression of proton-urate transporters. Our results showing the increased expression of NHE3 and URAT1 in the proximal tubules of THP mutant mice (Fig 8C and 8D) lend direct experimental support that this mechanism may indeed be operative in UAKD patients. Further studies are warranted to pinpoint the specific signals and pathways that mediate the responses between TAL and proximal tubule.

While our young THP mutant mice had polyuria, the old THP mutant mice were severely oliguric (Table 1; Fig 6). We believe that several factors played a part in this progressive phenotype including extensive interstitial fibrosis (Figs 4 and 5), loss of nephrons, and subsequent reduced glomerular filtration rate (Fig 6B). In this scenario the hypertrophied proximal tubules of remaining nephrons appear able to compensate for the ongoing pathologic naturesis in the TAL when overall glomerular filtration falls below a critical point (Table 1; Fig 6). While the old THP mutant mice have severe oliguria, we found that their water and food intake were much lower than age-matched wild-type littermates. This raises the interesting possibility that the old THP mutant mice are severely hypovolemic, resulting in highly concentrated urine. Further experiments are clearly warranted to test these possibilities. We had difficulty in following our THP mutant mice beyond 12-months because they began to succumb to deaths at this age, which is equivalent to a mid-aged person. Patients with UAKD also start to develop ESRD around this time [14,15].

Our current study may also have relevance to the previously reported associations between THP genetic variability and CKD risk. In data pooled from several large cohorts, SNPs within THP have associated with CKD risk [42,43]. These findings were also validated in an Icelandic cohort [44]. In this group the effect of THP genetic variability was additive with other CKD risk factors (e.g. age, diabetes, hypertension). Among those with disease-causing THP mutations, there is also evidence for a genotype-phenotype correlation. Mutations in the EGF domains have an earlier age to ESRD, while those in the cysteine-rich regions are latest and the D8C domain intermediate [45]. However, amongst patients with THP mutations, increased urinary excretion of THP precedes CKD. In human studies from a general population, urinary THP excretion does not clearly correlate with CKD risk [46]. Thus, it is clear much remains to be learned regarding the mechanisms of THP genotype-phenotype correlations in relationship to CKD, and use of mouse lines with targeted mutations could provide great insight.

Besides serving as a tool for better understanding the molecular pathogenesis of UAKD, THP mutant transgenic mice should be useful for evaluating new therapeutics that improve the folding of THP mutants. As we showed previously with THP mutants expressed in culture cells, certain chemicals, in particular sodium 4-phenylbutyrate and probenecid, markedly reduced ER retention of the C217G THP mutant and increased its surface translocation and secretion into the culture media [21]. It would be of considerable interest and importance to test whether similar effects could be produced in the transgenic mice, thus paving the way for potential application in humans. Other approaches including chemical chaperones might also have potential to partially or completely rescue the THP-mutation-caused phenotype.

## References

[1] Kumar S, Muchmore A (1990) Tamm-Horsfall protein—uromodulin (1950–1990). Kidney Int 37: 1395–1401. 2194064 10.1038/ki.1990.128

[2] Serafini-Cessi F, Malagolini N, Cavallone D (2003) Tamm-Horsfall glycoprotein: biology and clinical relevance. Am J Kidney Dis 42: 658–676. 14520616 10.1016/s0272-6386(03)00829-1

[3] Rampoldi L, Scolari F, Amoroso A, Ghiggeri G, Devuyst O (2011) The rediscovery of uromodulin (Tamm-Horsfall protein): from tubulointerstitial nephropathy to chronic kidney disease. Kidney Int 80: 338–347. doi: 10.1038/ki.2011.134 21654721 10.1038/ki.2011.134

[4] El-Achkar TM, Wu XR (2012) Uromodulin in kidney injury: an instigator, bystander, or protector? Am J Kidney Dis 59: 452–461. doi: 10.1053/j.ajkd.2011.10.054 22277744 10.1053/j.ajkd.2011.10.054 PMC3288726

[5] Sikri KL, Foster CL, Bloomfield FJ, Marshall RD (1979) Localization by immunofluorescence and by light- and electron-microscopic immunoperoxidase techniques of Tamm-Horsfall glycoprotein in adult hamster kidney. Biochem J 181: 525–532. 391220 10.1042/bj1810525 PMC1161191

[6] Khan SR, Kok DJ (2004) Modulators of urinary stone formation. Front Biosci 9: 1450–1482. 14977559 10.2741/1347

[7] Hart TC, Gorry MC, Hart PS, Woodard AS, Shihabi Z, Sandhu J, et al (2002) Mutations of the UMOD gene are responsible for medullary cystic kidney disease 2 and familial juvenile hyperuricaemic nephropathy. J Med Genet 39: 882–892. doi: 10.1136/jmg.39.12.882 12471200 10.1136/jmg.39.12.882 PMC1757206

[8] Bleyer AJ, Zivna M, Kmoch S (2011) Uromodulin-associated kidney disease. Nephron Clin Pract 118: c31–36. doi: 10.1159/000320889 21071970 10.1159/000320889

[9] Scolari F, Caridi G, Rampoldi L, Tardanico R, Izzi C, Pirulli D, et al (2004) Uromodulin storage diseases: clinical aspects and mechanisms. Am J Kidney Dis 44: 987–999. 15558519 10.1053/j.ajkd.2004.08.021

[10] Cameron JS, Simmonds HA (2005) Hereditary hyperuricemia and renal disease. Semin Nephrol 25: 9–18. 15660329 10.1016/j.semnephrol.2004.09.003

[11] Lhotta K (2010) Uromodulin and chronic kidney disease. Kidney Blood Press Res 33: 393–398. doi: 10.1159/000320681 20948228 10.1159/000320681

[12] Capasso G, Jaeger P, Robertson WG, Unwin RJ (2005) Uric acid and the kidney: urate transport, stone disease and progressive renal failure. Curr Pharm Des 11: 4153–4159. 16375737 10.2174/138161205774913219

[13] Bleyer AJ, Kmoch S (2016) Tamm Horsfall Glycoprotein and Uromodulin: It Is All about the Tubules! Clin J Am Soc Nephrol 11: 6–8. doi: 10.2215/CJN.12201115 26683889 10.2215/CJN.12201115 PMC4702239

[14] Scolari F, Ghiggeri GM (2003) Nephronophthisis-medullary cystic kidney disease: from bedside to bench and back again. Saudi J Kidney Dis Transpl 14: 316–327. 17657103

[15] Iorember FM, Vehaskari VM (2014) Uromodulin: old friend with new roles in health and disease. Pediatr Nephrol 29: 1151–1158. doi: 10.1007/s00467-013-2563-z 23880785 10.1007/s00467-013-2563-z

[16] Jennings P, Aydin S, Kotanko P, Lechner J, Lhotta K, Williams S, et al (2007) Membrane targeting and secretion of mutant uromodulin in familial juvenile hyperuricemic nephropathy. J Am Soc Nephrol 18: 264–273. doi: 10.1681/ASN.2006020158 17151335 10.1681/ASN.2006020158

[17] Bernascone I, Vavassori S, Di Pentima A, Santambrogio S, Lamorte G, Amoroso A, et al (2006) Defective intracellular trafficking of uromodulin mutant isoforms. Traffic 7: 1567–1579. doi: 10.1111/j.1600-0854.2006.00481.x 17010121 10.1111/j.1600-0854.2006.00481.x

[18] Vylet'al P, Kublova M, Kalbacova M, Hodanova K, Baresova V, Stibůrková B, et al (2006) Alterations of uromodulin biology: a common denominator of the genetically heterogeneous FJHN/MCKD syndrome. Kidney Int 70: 1155–1169. doi: 10.1038/sj.ki.5001728 16883323 10.1038/sj.ki.5001728

[19] Williams SE, Reed AA, Galvanovskis J, Antignac C, Goodship T, Karet FE, et al (2009) Uromodulin mutations causing familial juvenile hyperuricaemic nephropathy lead to protein maturation defects and retention in the endoplasmic reticulum. Hum Mol Genet 18: 2963–2974. doi: 10.1093/hmg/ddp235 19465746 10.1093/hmg/ddp235 PMC2714724

[20] Yang H, Wu C, Zhao S, Guo J (2004) Identification and characterization of D8C, a novel domain present in liver-specific LZP, uromodulin and glycoprotein 2, mutated in familial juvenile hyperuricaemic nephropathy. FEBS Lett 578: 236–238. doi: 10.1016/j.febslet.2004.10.092 15589826 10.1016/j.febslet.2004.10.092

[21] Ma L, Liu Y, El-Achkar TM, Wu XR (2012) Molecular and cellular effects of Tamm-Horsfall protein mutations and their rescue by chemical chaperones. J Biol Chem 287: 1290–1305. doi: 10.1074/jbc.M111.283036 22117067 10.1074/jbc.M111.283036 PMC3256885

[22] Takiue Y, Hosoyamada M, Yokoo T, Kimura M, Ochiai M, Kaneko K, et al (2008) Production and characterization of transgenic mice harboring mutant human UMOD gene. Nucleosides Nucleotides Nucleic Acids 27: 596–600. doi: 10.1080/15257770802136065 18600511 10.1080/15257770802136065

[23] Bernascone I, Janas S, Ikehata M, Trudu M, Corbelli A, Schaeffer C, et al (2010) A transgenic mouse model for uromodulin-associated kidney diseases shows specific tubulo-interstitial damage, urinary concentrating defect and renal failure. Hum Mol Genet 19: 2998–3010. doi: 10.1093/hmg/ddq205 20472742 10.1093/hmg/ddq205

[24] Wu X, Wakamiya M, Vaishnav S, Geske R, Montgomery C Jr., Jones P, et al (1994) Hyperuricemia and urate nephropathy in urate oxidase-deficient mice. Proc Natl Acad Sci U S A 91: 742–746. 8290593 10.1073/pnas.91.2.742 PMC43025

[25] Palmer BF (2011) Metabolic complications associated with use of diuretics. Semin Nephrol 31: 542–552. doi: 10.1016/j.semnephrol.2011.09.009 22099511 10.1016/j.semnephrol.2011.09.009

[26] Hediger MA, Johnson RJ, Miyazaki H, Endou H (2005) Molecular physiology of urate transport. Physiology (Bethesda) 20: 125–133. 15772301 10.1152/physiol.00039.2004

[27] Rafey MA, Lipkowitz MS, Leal-Pinto E, Abramson RG (2003) Uric acid transport. Curr Opin Nephrol Hypertens 12: 511–516. 12920398 10.1097/00041552-200309000-00005

[28] Zhu X, Cheng J, Gao J, Lepor H, Zhang ZT, Pak J, et al (2002) Isolation of mouse THP gene promoter and demonstration of its kidney-specific activity in transgenic mice. Am J Physiol Renal Physiol 282: F608–617. doi: 10.1152/ajprenal.00297.2001 11880321 10.1152/ajprenal.00297.2001

[29] Zhu X, Cheng J, Huang L, Gao J, Zhang ZT, Pak J, et al (2003) Renal tubule-specific expression and urinary secretion of human growth hormone: a kidney-based transgenic bioreactor. Transgenic Res 12: 155–162. 12739883 10.1023/a:1022967505222

[30] Muratsubaki H, Satake K, Enomoto K (2006) Enzymatic assay of allantoin in serum using allantoinase and allantoate amidohydrolase. Anal Biochem 359: 161–166. doi: 10.1016/j.ab.2006.09.024 17081493 10.1016/j.ab.2006.09.024

[31] Turner JJ, Stacey JM, Harding B, Kotanko P, Lhotta K, Puig JG, et al (2003) UROMODULIN mutations cause familial juvenile hyperuricemic nephropathy. J Clin Endocrinol Metab 88: 1398–1401. doi: 10.1210/jc.2002-021973 12629136 10.1210/jc.2002-021973

[32] Hession C, Decker JM, Sherblom AP, Kumar S, Yue CC, Mattaliano RJ, et al (1987) Uromodulin (Tamm-Horsfall glycoprotein): a renal ligand for lymphokines. Science 237: 1479–1484. 3498215 10.1126/science.3498215

[33] Lavrik IN, Golks A, Krammer PH (2005) Caspases: pharmacological manipulation of cell death. J Clin Invest 115: 2665–2672. doi: 10.1172/JCI26252 16200200 10.1172/JCI26252 PMC1236692

[34] Castrop H, Schiessl IM (2014) Physiology and pathophysiology of the renal Na-K-2Cl cotransporter (NKCC2). Am J Physiol Renal Physiol 307: F991–f1002. doi: 10.1152/ajprenal.00432.2014 25186299 10.1152/ajprenal.00432.2014

[35] Haas M, Forbush B 3rd (1998) The Na-K-Cl cotransporters. J Bioenerg Biomembr 30: 161–172. 9672238 10.1023/a:1020521308985

[36] Renigunta A, Renigunta V, Saritas T, Decher N, Mutig K, Waldegger S. (2011) Tamm-Horsfall glycoprotein interacts with renal outer medullary potassium channel ROMK2 and regulates its function. J Biol Chem 286: 2224–2235. doi: 10.1074/jbc.M110.149880 21081491 10.1074/jbc.M110.149880 PMC3023518

[37] Anzai N, Jutabha P, Amonpatumrat-Takahashi S, Sakurai H (2012) Recent advances in renal urate transport: characterization of candidate transporters indicated by genome-wide association studies. Clin Exp Nephrol 16: 89–95. doi: 10.1007/s10157-011-0532-z 22038265 10.1007/s10157-011-0532-z

[38] Enomoto A, Endou H (2005) Roles of organic anion transporters (OATs) and a urate transporter (URAT1) in the pathophysiology of human disease. Clin Exp Nephrol 9: 195–205. doi: 10.1007/s10157-005-0368-5 16189627 10.1007/s10157-005-0368-5

[39] Donowitz M, Li X (2007) Regulatory binding partners and complexes of NHE3. Physiol Rev 87: 825–872. doi: 10.1152/physrev.00030.2006 17615390 10.1152/physrev.00030.2006

[40] Schaeffer C, Cattaneo A, Trudu M, Santambrogio S, Bernascone I, Giachino D, et al (2012) Urinary secretion and extracellular aggregation of mutant uromodulin isoforms. Kidney Int 81: 769–778. doi: 10.1038/ki.2011.456 22237754 10.1038/ki.2011.456

[41] Mutig K, Kahl T, Saritas T, Godes M, Persson P, Bates J, et al (2011) Activation of the bumetanide-sensitive Na+,K+,2Cl- cotransporter (NKCC2) is facilitated by Tamm-Horsfall protein in a chloride-sensitive manner. J Biol Chem 286: 30200–30210. doi: 10.1074/jbc.M111.222968 21737451 10.1074/jbc.M111.222968 PMC3191059

[42] Kottgen A, Glazer NL, Dehghan A, Hwang SJ, Katz R, Li M, et al (2009) Multiple loci associated with indices of renal function and chronic kidney disease. Nat Genet 41: 712–717. doi: 10.1038/ng.377 19430482 10.1038/ng.377 PMC3039280

[43] Kottgen A, Hwang SJ, Larson MG, Van Eyk JE, Fu Q, Benjamin EJ, et al (2010) Uromodulin levels associate with a common UMOD variant and risk for incident CKD. J Am Soc Nephrol 21: 337–344. doi: 10.1681/ASN.2009070725 19959715 10.1681/ASN.2009070725 PMC2834540

[44] Gudbjartsson DF, Holm H, Indridason OS, Thorleifsson G, Edvardsson V, Sulem P, et al (2010) Association of variants at UMOD with chronic kidney disease and kidney stones-role of age and comorbid diseases. PLoS Genet 6: e1001039 doi: 10.1371/journal.pgen.1001039 20686651 10.1371/journal.pgen.1001039 PMC2912386

[45] Moskowitz JL, Piret SE, Lhotta K, Kitzler TM, Tashman AP, Velez E, et al (2013) Association between genotype and phenotype in uromodulin-associated kidney disease. Clin J Am Soc Nephrol 8: 1349–1357. doi: 10.2215/CJN.11151012 23723338 10.2215/CJN.11151012 PMC3731914

[46] Shlipak MG, Li Y, Fox C, Coresh J, Grunfeld C, Whooley M. (2011) Uromodulin concentrations are not associated with incident CKD among persons with coronary artery disease. BMC Nephrol 12: 2 doi: 10.1186/1471-2369-12-2 21235779 10.1186/1471-2369-12-2 PMC3031204

